# Review of the Efficacy and Safety of Gadopiclenol: A Newly Emerging Gadolinium-Based Contrast Agent

**DOI:** 10.7759/cureus.43055

**Published:** 2023-08-06

**Authors:** Emad Alsogati, Hussain Ghandourah, Amal Bakhsh

**Affiliations:** 1 Department of Radiology, King Fahd General Hospital, Jeddah, SAU

**Keywords:** relaxivity, safety, gadolinium-based contrast agent, magnetic resonance imaging, gadopiclenol

## Abstract

Gadolinium-based contrast agents (GBCAs) are one of the most commonly used agents in magnetic resonance imaging. Gadopiclenol is a new GBCA aimed at providing improved diagnostic efficacy with a favorable safety profile. The proposed advantages are due to its specific pharmacological properties, one of which is high relaxivity values. The aim of this review is to assess the efficacy, diagnostic accuracy, and safety of gadopiclenol in comparison to other currently used gadolinium-based contrast agents. PubMed and other database systems were used to identify relevant studies. The Preferred Reporting Items for Systematic Reviews and Meta-analysis (PRISMA) guidelines were followed, resulting in 10 articles that were included in the review. The outcomes were reviewed according to several factors regarding efficacy and accuracy in terms of qualitative and quantitative descriptors relative to properties of enhancement provided by the contrast agent. In terms of safety profile, a number of outcomes were assessed such as the occurrence of serious adverse effects, severe kidney injury, and organ-based contrast retention. Gadopiclenol was found to provide outcomes comparable to other commonly used GBCAs at lower doses with further favorable results at higher doses while maintaining an acceptable safety profile. However, it was found to have high rates of retention within the liver and can cause nonsignificant QT prolongation in healthy individuals, which arguably creates the need for further research regarding more long-term implications of these possible adverse effects.

## Introduction and background

The most commonly used contrast agents in current times include gadolinium-based contrast agents (GBCAs). GBCAs have revolutionized modern technological advances in radiological diagnostics. Its implementation in magnetic resonance imaging (MRI) has pioneered innovative techniques in pathology detection most notably in the central nervous system (CNS). Pathology ranges from vascular to white matter disease and oncologic tumors. The use of GBCA has increased diagnostic accuracy in detecting minute lesions and improved CNS tissue visualization through enhanced characterization of several distinct CNS pathologies. It helps differentiate between neoplastic pathology and white matter disease entities [1].

Use of contrast media has shown growing concern among the Food and Drug Agencies of multiple countries worldwide due to its risk of causing renal disease and risk of tissue deposition [2]. Contrast has been found to mainly deposit within multiple bodily tissues, most commonly within the central nervous system. The current most commonly used and approved GBCAs can be divided into linear or macrocyclic categories. Linear GBCAs have been found to be associated with renal impairment likely due to deposition in renal parenchymal tissue [3-4]. The most worrying renal adverse effect concerns nephrogenic systemic fibrosis (NSF), which involves diffuse fibrotic changes involving multiple organs [3]. Linear GBCAs have also been found to deposit in CNS and bone tissue, which ultimately led to the prohibition of its use in Europe apart from MRI contrast-enhanced studies utilizing certain specific linear GBCAs namely gadobenic acid and gadoxetate disodium [5-8]. Restricted use of linear GBCAs has also been implemented in Japan and the US with an overall consensus of using as low as possible GBCA concentrations to prevent possible adverse effects [2]. The higher the dosage of contrast, the better the diagnostic accuracy, however, the highest acceptable contrast dosage is 0.1 mmol/kg body weight. A solution to having limited diagnostic accuracy while maintaining optimal adverse risk prevention is using GBCAs with high T1-weighted relaxivity. Higher T1-relaxivity is achieved by using higher molecular weight molecules. A downside of using such molecules is that it leads to low distribution volume which decreases tumor enhancement on MRI [9-10].

Gadopiclenol is a newly developed GBCA known to have high relaxivity and high kinetic stability in an acidic environment compared to other conventionally used GBCAs such as gadoterate, gadobutrol, gadodiamide, and gadopentetate, which decreases the risk of gadolinium accumulation [11]. Known to have a macrocyclic ligand structure, gadopiclenol has a low molecular weight and demonstrates a lack of interaction with plasma proteins, which renders it favorable for several clinical indications including lesions of the CNS and breast cancer. High solubility and low osmolality in water are some of its desirable physicochemical properties [12].

The aim of this review is to assess gadopiclenol in terms of efficacy, diagnostic accuracy, and safety.

## Review

Methods

The literature search was performed at multiple time points during the study to incorporate any possible new published material, with the most recent search being performed on July 1, 2023. The search was conducted using PubMed, Medline, and Web of Science databases to identify all relevant articles concerning the new GBCA “Gadopiclenol” using appropriate MESH and relative terms. A specific search strategy was developed to dictate the inclusion and exclusion criteria of the yielded articles. Randomized control trials (RCT), animal studies, and other clinical studies were included. Purely pharmacological studies were excluded as direct assessment of the pharmacologic properties of this new contrast agent was not the objective of this study. Other review articles were also excluded. Due to the narrow scope of this topic, no other restrictions were applied.

**Figure 1:**
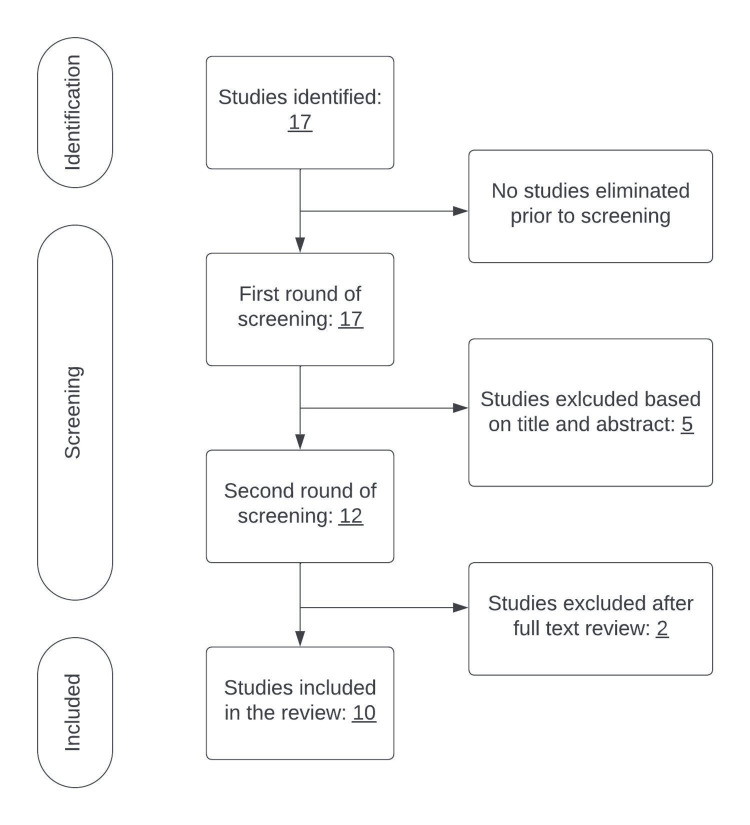
PRISMA flow diagram demonstrating the search strategy.

The resulting abstracts were analyzed objectively according to the relevance in concurrence with the aim of this study undergoing a first round of exclusion and a second round of exclusion was undergone by screening the provided full-text. Both rounds were performed by the same two reviewers HG and AB. No major discrepancies occurred during these processes. The search ultimately yielded 10 articles to be included in the review (Table 1). The process is demonstrated in the Preferred Reporting Items for Systematic Reviews and Meta-analysis (PRISMA) guidelines flow diagram (Figure 1).

**Table 1:** Studies included in the review ^1 ^The study included three sub-studies and assessed six rats in the first study, 12 rats in the second study and 30 rats in the third study. ^2^ Total number of included participants was 256, however, 14 discontinued the study creating a total of 242 participants.

Reference	Type of study	Sample size	Comparison	Relevant outcomes
Bendszus M et al. [11]	Phase IIb double-blind randomized controlled trial	272	Gadobenate dimeglumine	Efficacy and safety
Robert P et al. [13]	Animal study	32	Gadoterate meglumine, Gadobutrol, & Gadobenate dimeglumine	Efficacy
Fries P et al. [14]	Animal study	20	Gadopentetate & Gadoterate	Efficacy
Violas X et al. [15]	Animal study	48 ^1^	Gadobenate dimeglumine	Efficacy
Hao J et al. [16]	Phase I/IIa study	54	Placebo	Efficacy and safety
Loevner LA et al. [17]	Double-blind randomized controlled trial	256 ^2^	Gadobutrol	Efficacy and safety
Funck-Brentano C et al. [18]	Double-blind randomized controlled trial	48	Placebo	Safety
Fretellier N et al. [19]	Animal study	40	Gadoterate meglumine, Gadodiamide & Gadobutrol	Safety
Jurkiewicz E et al. [20]	Phase II study	80	NA	Safety
Bradu A et al. [21]	Phase I study	40	NA	Safety

Possible reporting bias, certainty assessment, and effect measure were not addressed in this review. Assessment of possible causes for heterogeneity or sensitivity analysis of the provided results was not performed in this study.

Results

The results can be categorized under two main groups, diagnostic accuracy and efficacy in terms of quantitative and qualitative assessments and safety profiles.

Articles discussing qualitative outcomes included factors that describe the appearance of the target-enhanced lesions. These parameters include border delineation which describes the conspicuity and definition of the lesion in relation to surrounding normal tissue, internal morphology which describes the internal structure of the lesion which can be heterogenous or homogenous, and overall contrast enhancement. Quantitative assessments included contrast-noise ratio (CNR), signal-noise ratio (SNR), contrast enhancement percentage, and overall number of enhancing lesions detected. SNR values describe the effect of noise on desired target tissue signal intensity which reflects image quality [22]. CNR describes the difference in signal intensity produced by two different paramagnetic structures and compares the signal intensity produced by contrast in different sequences [23]. Both SNR and CNR can affect the quality of images obtained which helps facilitate radiologists [24].

The safety outcomes included the occurrence and intensity of adverse events. Specific adverse reactions such as the possible occurrence of NSF or prolonged cardiac QT intervals were taken into more detailed consideration within a few articles.

Diagnostic accuracy and efficacy

Six studies assessed contrast performance with outcomes relevant to this review and five of which performed a direct comparison with other GBCAs. A large double-blinded RCT assessed the efficacy of gadopiclenol under qualitative and quantitative parameters using gadobenate dimeglumine as a direct comparison with the help of three participating readers [11]. Gadobenate dimeglumine is a conventionally used GBCA for CNS imaging known for its high relaxivity times. CNR parameters were the primary quantitative outcome for this study, where gadopiclenol was found to have statistically significant higher CNR values at 0.1 and 0.2 mmol/kg in comparison to 0.1 mmol/kg of gadobenate dimeglumine and similar performance at 0.05 mmol/kg. Lesion-to-brain ratios and contrast enhancement percentage were secondary outcomes and showed similar results. The most enhancing lesions that were free from artifacts underwent qualitative assessment where the gadopiclenol group showed similarly higher lesion internal morphology and border delineation parameters. The mean scores were again overall higher for gadopiclenol at 0.1 and 0.2 mmol/kg showing statistical significance. Overall gadopiclenol was preferred to gadobenate dimeglumine at 0.1 and 0.2 mmol/kg by the participating readers with no preference reported at 0.05 mmol/kg dosage. Moreover, no significant change in relaxivity was noted using gadopiclenol at 1.5 and 3 T magnetic field strengths which helps preserve the high quality of enhancement provided by the contrast agent [11].

An animal study compared gadopiclenol at multiple doses to three reference GBCAs namely gadoterate meglumine, gadobutrol, and gadobenate dimeglumine using brain glioma-induced rats [13]. The qualitative assessment included lesion internal morphology, border delineation, and contrast enhancement. Gadopiclenol at 0.05 mmol/kg and higher was found to be superior to the other three GBCAs at 0.1 mmol/kg in these parameters with the results being graded as good to excellent. Additionally, gadopiclenol at 0.1 and 0.2 mmol/kg showed increased clarity of more complex tumors providing more unique characteristics to these lesions that were not identified in the comparative GBCAs. In terms of diagnostic preference, gadopiclenol at 0.05 and 0.075 mmol/kg was described as mostly equal or superior to the other three GBCAs. For 0.1 and 0.2 mmol/kg, gadopiclenol was always preferred over the other GBCAs by all readers. For quantitative analysis, 0.05 mmol/kg of gadopiclenol did not show a significant difference in CNR values in comparison to the reference GBCAs; however, it showed statistically significant favorable results at 0.1 mmol/kg with almost double CNR higher than the reference GBCAs and even further at 0.2 mmol/kg dosage [13].

In another animal study, gadopiclenol was compared to reference GBCAs, namely gadopentetate and gadoterate at 0.1 mmol/kg dosage, using tumor-induced rats specifically targeting liver tissue. In-vivo and in-vitro MRI assessments were performed and relaxivity times were assessed at different magnetic field strengths [14]. Quantitative criterions included CNR, SNR, and lesion enhancement (LE). Similar to the previous studies, gadopiclenol was found to have statistically significant higher and more than double SNR, CNR, and LE values in normal liver tissue and liver tumors in comparison to the reference GBCAs at all concentrations, further demonstrating its advantages. Gadoterate and gadopentetate showed decreasing relaxivity across higher magnetic field strengths. In comparison, gadopiclenol showed overall higher relaxivity times between increasing magnetic field strengths from 1.5 to 9.4 T with less reduction in relaxivity and provided more favorable contrast-enhanced images at higher strengths [14].

One animal study compared gadopiclenol to gadobenate dimeglumine in rats containing induced small brain lesions to assess enhancement as well as to assess fourth ventricle enhancement through quantitative assessment [15]. Brain lesion enhancement was assessed by the number of enhancing voxels present in comparison to healthy brain tissue using doses of 0.1 mmol/kg for both contrast agents. Fourth ventricle enhancement was measured by an increase in SNR compared to baseline values extracted from preinjected tissue using a high dose of 1.2 mmol/kg for maximum enhancement purposes to allow for better detection. The gadopiclenol group showed statistically significant better enhancement for both outcomes, with almost double enhancement noted in the fourth ventricles [15].

A phase I and phase IIa study assessed the performance of gadopiclenol in comparison to controlled-placebo at different doses with doses 0.05, 0.075, 0.1, and 0.2 mmol/kg concerning diagnostic accuracy and efficacy outcomes [16]. The study included test subjects with at least one previously detected brain lesion on MRI with respective disruption of the blood-brain-brain to allow lesion enhancement. Qualitative measures included an assessment of border delineation, internal morphology, and contrast enhancement brightness. A quantitative assessment included SNR, CNR, and overall signal intensity of the brain lesions. Both qualitative and quantitative outcomes showed improving outcomes with increasing doses of gadopiclenol used [16].

A recently published large international RCT named the PICTURE study assessed the outcomes of gadopiclenol in MRI studies [17]. The study similarly utilized qualitative assessment including border delineation, internal morphology, and contrast enhancement as well as quantitative assessment including CNR. Gadopiclenol at 0.05 mmol/kg concentration was compared to gadobutrol at 0.1 mmol/kg concentration and showed significantly similar results in terms of lesion border delineation, internal morphology, contrast enhancement, and CNR, concluding that gadopiclenol is not inferior to gadobutrol at half its normal concentration [17].

Safety profile

The incidence of adverse events (AEs) was assessed in seven studies. No significant AEs were reported as associated with gadopiclenol in two RCTs [16, 18]. The first RCT compared gadopiclenol to placebo and where the gadopiclenol resulted in only mild AEs mostly including pain and edema at the injection site as well as the occurrence of headaches [16]. Furthermore, no significant correlation between the dose of gadopiclenol and the incidence of AEs was detected. The second RCT compared the outcomes of gadopiclenol at a clinical dose of 0.1 mmol kg−1 and supraclinical dose of 0.3 mmol kg−1 to placebo and resulted in mostly mild AEs with only a few moderate reactions with no significant difference between the different groups [18]. The study further assessed cardiac safety in terms of QT prolongation by including participants free from cardiac disease history or possible risk factors for QT prolongation. Both doses of gadopiclenol were found to cause non-significant QT prolongation which was higher in the supraclinical dose group and resolved very quickly as well [18].

An animal study compared gadopiclenol to two conventionally used macrocyclic GBCAs gadoterate meglumine and gadobutrol and one linear GBCA namely gadodiamide in rats with clinically induced renal failure [19]. The study assessed possible gadolinium retention in organs through plasma mass spectroscopy and incidence of AEs focusing on possible NSF and dermatological lesions through histopathology and microscopic analysis. Firstly, total washout was successfully achieved in all GBCAs. Secondly, gadolinium retention was reported as significantly higher in the gadodiamide group within biopsied samples of skin, liver, renal, splenic, bone, and CNS tissue. All macrocyclic GBCAs showed non-significant gadolinium retention, however, significantly higher gadolinium retention within liver tissue was found in gadopiclenol in comparison. Finally, the incidence of AEs namely NSF-like manifestations and systemic toxicity were found in the gadodiamide group whereas none occurred in the remaining macrocyclic GBCA groups including gadopiclenol [19].

One previously described RCT found a possible positive correlation between the incidence of AEs and increasing dosage of administered gadopiclenol with only one serious adverse event (SAEs) occurring with gadopiclenol [11]. The SAE in question was an increase of creatinine levels above 25%, however, it resolved within 24 hours and was consequently described as clinically non-significant [11]. The remaining non-serious AEs related to gadopiclenol included injection-site pain, headache, injection-site coldness, fatigue, and diarrhea [11].

The PICTURE study assessed the safety profile of gadopiclenol as well and found only mild AEs occurring in 12 patients out of a total of 247 test subjects. The AEs were variable and nonspecific but were very similar to other mild reactions occurring in the comparison GBCA which has already established its overall safety. These reactions were not classified as serious and no further SAEs occurred within the gadopiclenol group [17].

An uncontrolled, international, and multi-center pediatric phase II study recruited volunteers aged 2-17 years who were suspected of or diagnosed with specific lesions and were already planned to undergo follow-up MRI scans [20]. The study reported only two AEs occurring in two patients that were considered associated with gadopiclenol administration. One was the incidence of a moderately intense maculopapular rash that resolved within seven days post-injection and the other concerned the incidence of a non-significant prolongation in cardiac QT time occurring in a five-year-old participant which did not resolve after the study and with no follow-up performed. Otherwise, no other significant AEs were reported [20].

A phase I study recruited healthy subjects and multiple renally impaired subjects of varying intensities including some patients with end-stage renal disease (ESRD) to assess the safety of gadopiclenol [21]. One of the aims of the study was to assess gadolinium elimination and the incidence of possible AEs in association with a single 0.1 mmol/kg dose of gadopiclenol administration. Firstly, there was decreased gadolinium elimination in more severely renally impaired subjects, however, total or near-total elimination was achieved in all subjects at the end of the study. Subjects with ESRD required up to three dialysis sessions with near-total significant elimination of systemic gadolinium. Secondly, only six AEs occurred in association with gadopiclenol administration which involved increased creatinine levels, however, the increase was considered non-significant and non-dangerous. Finally, no NSF manifestations occurred in any of the renally impaired subjects [21].

Discussion

On September 21, 2022, gadopiclenol (Elucirem^TM^) was approved by the FDA for use in the United States [25]. According to the results of this review, gadopiclenol has consistently shown improved diagnostic results through qualitative and quantitative assessments in comparison to conventionally used GBCAs. Contrast enhancement properties improved with increasing gadopiclenol concentrations and 0.05 mmol/kg of gadopiclenol was found to be considered equal to or superior to standard 0.1 mmol/kg of conventional GBCAs with more favorable preference by readers specifically using higher doses at 0.1 and 0.2 mmol/kg [11]. Therefore, in terms of efficacy gadopiclenol has demonstrated on par or greater performance at 0.05 mmol/kg concentration which is half of the standard dose of what conventional GBCAs are administered currently [11, 13, 17]. This would allow for more flexibility in using the regular clinical dose of 0.1 mmol/kg if better contrast enhancement is desired for more complex lesions. These quantitative and qualitative outcomes provide higher diagnostic accuracy and efficacy for contrast-enhanced MRI scans which help further optimize the studies for better evaluation by radiologists. This improves lesion detection and workup which facilitates a better diagnosis.

Gadopiclenol works through its extracellular fluid contrast-based properties where it visualizes abnormal lesions, specifically tumors, which is facilitated by their abnormal vascularity and increased retention of contrast material [26]. Gadopiclenol achieves enhanced contrast MRI images through its paramagnetic properties which affect the relaxivity times of protons of water molecules in the target tissue [27]. This produces different signal intensities as seen on an MRI scan allowing enhanced lesion visualization. As presented in the results, one of the main advantageous properties of gadopiclenol is its high relaxivity which shortens T1 relaxation time allowing for better contrast-enhanced images. This is facilitated by its pharmacodynamics and molecular structure through increased water protons interaction that is higher than other GBCAs such as gadopentetate or gadoterate [12, 14]. Within the established group of commonly marketed GBCAs, gadobutrol is a macrocyclic GBCA with the current highest relaxivity which is why it was chosen as a reference in the PICTURE randomized clinical trial [12, 17]. Furthermore, relaxivity times have been found to deteriorate with increasing magnetic field strength in GBCAs [28]. Compounds with relatively low molecular weights such as conventionally used GBCAs gadoterate or gadopentetate provide high relaxivity times that are less susceptible to increasing magnetic field strengths which is one of their utilized advantages [14]. However, in comparison, gadopiclenol shows even less reduction in relaxivity times thus providing overall significantly higher relaxivity rates through varying magnetic field strengths with increased contrast potency [14]. Its relaxivity times are comparatively less susceptible to increasing magnetic field strength allowing it to be used in ultra-high field strengths for providing higher-quality images [13-16]. This helps improve diagnostic accuracy without the need to increase dosages. It can also potentially be used in dynamic contrast-enhanced MRI modalities such as cardiovascular imaging due to its pharmacokinetics and biodistribution properties [14]. However, there are no published studies regarding the performance of gadopiclenol in MR angiograms or perfusion imaging as of yet. This allows for the opportunity for further research to assess its feasibility in rapidly expanding fields such as cardiovascular imaging.

The main safety risk related to GBCA exposure is the development of serious AEs. As described earlier, only two instances where a serious AE occurred as previously described, namely QT prolongation and development of a moderately intense maculopapular rash. No other serious reaction occurred which could be attributed to the administration of gadopiclenol. In terms of gadolinium retention, gadopiclenol showed no significant retention within the different biopsied tissue samples except for liver parenchyma, however, without the presence of any liver abnormalities being detected. This was hypothesized by the authors as possibly due to its higher fecal excretion rates within renally impaired rats with decreased urinary excretion which could be due to the specific chemical structure of gadopiclenol [19]. Nonetheless, additional long-term phase III and IV trials with possible repetitive administration of the contrast agent are required to study possible accumulation in liver tissue and fully assess its safety profile regarding gadolinium tissue retention and possible associated long-term effects. 

An additional main concern is the development of NSF which develops in patients suffering from pre-existing renal impairment [29, 30]. It is rare and potentially dangerous, however, it is mainly associated with linear GBCAs rather than macrocyclic agents like gadopiclenol [20]. Nephrogenic systemic fibrosis did not occur in any of the studies, most notably within the previously described study that recruited different patients with varying intensities of pre-existing renal impairment which included patients with ESRD requiring dialysis after gadopiclenol administration [21]. No NSF had occurred and almost all gadopiclenol concentrations were cleared following three dialysis sessions, however, this study assessed the outcome of NSF only within a short-term follow-up period of six months. Furthermore, the possible long-term effects of cumulative repeated doses of gadopiclenol are not fully known. The possible occurrence of NSF in the long term over a period of years and from possible repeated exposure should be assessed further in order to provide a comprehensive outlook regarding its safety.

Cardiac QT intervals were assessed in detail by one of the studies and showed no significant QT prolongation [18]. However, this study included healthy volunteers with no cardiac illnesses, no history of cardiac disease, or use of medication that could possibly affect QT times. Therefore, clinically significant QT prolongation in patients who suffer from cardiac disease or take medications with possible side effects concerning QT time cannot be excluded. Furthermore, there is little research regarding the possible effects of GBCA on QT prolongation which could perhaps be an area of further research [18].

In order to obtain better images, enhancement can be improved by increasing contrast dosage or magnetic field strength [31, 32]. Increasing dosage introduces the risk of serious adverse reactions, whereas increasing magnetic field strength can affect relaxivity times influencing enhancement and contrast performance. Currently all conventionally used GBCAs have similar relaxivity times and risk of adverse events causing them to be used with caution [33]. Therefore, the entry of gadopiclenol provides approximately two-three times higher relaxivity facilitating improved images at relatively lower doses than currently used GBCAs [12]. The results of this review show overall significantly improved diagnostic accuracy in comparison to conventional GBCAs and demonstrated lower safety risks associated with gadopiclenol with the use of subclinical doses of 0.05 mmol/kg or otherwise a similar safety profile with the 0.1 mmol/kg dosage with no reported occurrence of any major or specific AEs.

## Conclusions

Gadopiclenol is a new macrocyclic GBCA with favorable relaxivity properties. Its specific pharmacological properties offer improved contrast enhancement with higher diagnostic accuracy and efficacy at a relatively lower dosage of 0.05 mmol/kg than currently used GBCAs. This leads to improved lesion detection and characterization by radiologists, ultimately leading to a more accurate diagnosis. It further provides significantly more favorable outcomes at equal doses of 0.1 mmol/kg. This allows for minimal safety risks without sacrificing desired diagnostic outcomes. Although it was found that gadopiclenol has some minor non-serious AEs, the benefits outweigh the risks. Further future research could be performed regarding these effects in order to fully assess its safety profile.
